# Voltage-Tunable Mid- and Long-Wavelength Dual-Band Infrared Photodetector Based on Hybrid Self-Assembled and Sub-Monolayer Quantum Dots

**DOI:** 10.3390/mi10010004

**Published:** 2018-12-22

**Authors:** Yao Zhai, Guiru Gu, Xuejun Lu

**Affiliations:** 1 Department of Electrical and Computer Engineering, University of Massachusetts Lowell, One University Avenue, Lowell, MA 01854, USA; Yao.Zhai@colorado.edu; 2 Department of Physics, Stonehill College, 320 Washington Street, Easton, MA 02357, USA; ggu@stonehill.edu

**Keywords:** submonolayer quantum dots, self-assembled quantum dot, dual-band midwave and longwave infrared photodetector, voltage tunable detection spectrum

## Abstract

In this paper, we report a mid-wave infrared (MWIR) and long-wave infrared (LWIR) dual-band photodetector capable of voltage-controllable detection band selection. The voltage-tunable dual-band photodetector is based on the multiple stacks of sub-monolayer (SML) quantum dots (QDs) and self-assembled QDs. By changing the photodetector bias voltages, one can set the detection band to be MWIR, or LWIR or both with high photodetectivity and low crosstalk between the bands.

## 1. Introduction

Due to their importance in numerous civilian and defense applications, dual-band photodetectors covering the mid-wave infrared (MWIR) (3–5 µm) and long-wave infrared (LWIR) (8–12 µm) bands have been extensively researched in the past decades [1,2]. The current state-of-the-art infrared detection technology is based on the HgCdTe alloy. By modifying Hg to Cd component ratio in the alloy [3], one can tune the detection spectrum from 1 to 25 µm. However, due to the difficulties of growing large size HgCdTe wafers with high quality and good uniformity, HgCdTe based IR photodetectors and focal plane arrays (FPAs) are not only very expensive and but also very hard to make, especially for large format (1024 × 1024) FPAs. Graphene photodetectors are a new emerging technology that could offer broadband detection, high responsivity, and high operating temperatures [4]. One of the limitations, however, is the availability of large area graphene for large format (1024 × 1024) FPA development. 

Quantum-dot infrared photodetector (QDIP) technology has been extensively studied as a promising large area IR sensing and imaging technology due to the advantages provided by the Quantum-dot (QD) nanostructures, including the three-dimensional (3D) quantum confinement that modifies the quantum selection rules to allow surface normal incident light detection [5], low dark current [6], high photoresponsivity [7], and high operating temperature [7,8,9]. QD based MWIR and LWIR dual-band photodetectors have been reported by several research groups [10,11,12] using different techniques including tailoring the sizes of the QDs during the material growth capping the QDs with different capping layers [12], and inserting asymmetric barrier layers [13]. Most of these dual band QDIPs are based on self-assembled QDs grew via the Stranski-Krastanow (S-K) growth mode (referred to as the self-assembled QDs henceforth). Submonolayer (SML) QD growth is an alternative QD growth mode [14,15,16], where only a fraction of a monolayer (ML) of InAs is grown followed by the growth of the GaAs separation layer. QDs form by growing multiple repeats of the SML InAs with the GaAs separation layer (referred to as the SML QDs henceforth) [14,15,16]. SML based QDs offer a few advantages, including smaller base width and absence of wetting layer, large density of QDs, reduced strain for more QD layers, and engineerable correlation between the vertically stacked QD layers by varying the thickness of the separation layers [15,17,18]. In addition, since the thicknesses of the SML InAs layer and its GaAs separation layer can be adjusted individually, the SML QD technique provides additional flexibility in designing the detection spectrum for multispectral sensing and imaging. Due to these advantages, SML QD based optoelectronic devices have been extensively researched, including lasers [16] and infrared (IR) photodetectors [11,17]. 

In this paper, we report a dual-band MWIR and LWIR photodetector based on the multiple stacks of SML QDs and self-assembled QDs. By engineering the SML and the self-assembled QDs, we obtained MWIR and LWIR dual-band detection with low crosstalk between the MWIR and the LWIR bands. The MWIR and LWIR dual-band photodetector also shows voltage-controllable band selection. By changing the photodetector bias voltages, one can set the detection band to be MWIR, or LWIR or both with low crosstalk and high photodetectivity.

## 2. Device Structure

**Figure 1:**
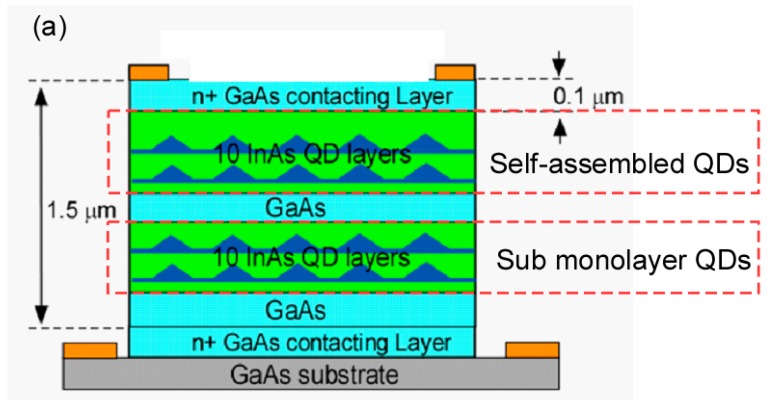

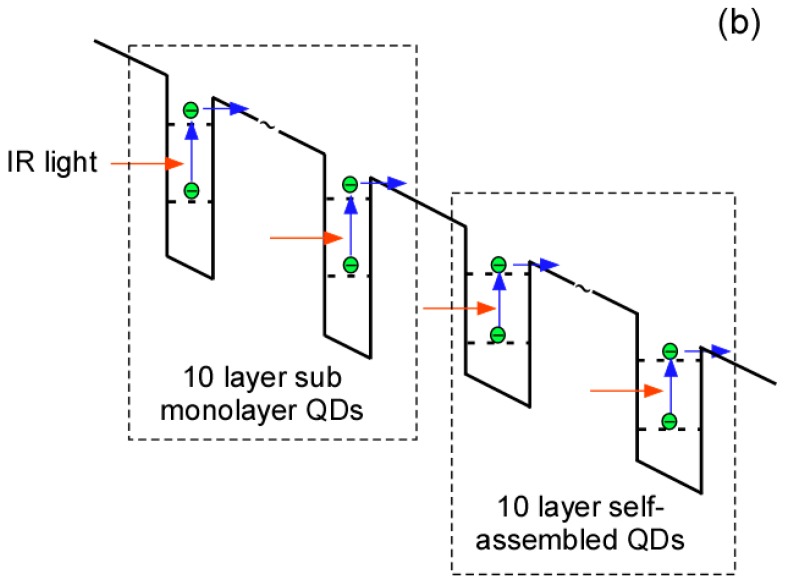
(**a**) Schematic structures of the hybrid Quantum-dot infrared photodetector (QDIP). It consists of the Submonolayer (SML) QDs and self-assembled QDs sandwiched between the top and the bottom contacts. (**b**) Simplified conduction band structure of the SML and self-assembled QDIP. Due to the different energy levels, the SML and self-assembled QDs can detect different bands.

Figure 1a shows the schematic structure of the hybrid QDIP. It consists of 10 layers of SML QDs and 10 layers of self-assembled QDs sandwiched between the top and bottom contact layers. The total thickness of the SML and the self-assembled QD layers is 1.5 μm. Figure 1b shows the simplified conduction band structure of the hybrid SML and the self-assembled QDIP. The QDIP is a photoconductor where the MWIR/LWIR incident light excites the electrons from the ground states to the excited states. The electrons are subsequently collected by the top and bottom electrodes and form photocurrent. Due to different strains in forming the SML QDs and self-assembled QDs, their sizes and the energy levels are unalike and thus allow them to detect different bands. By optimizing the QD growth conditions such as the growth rate, substrate temperatures, and the V to III ratios, one can achieve the detection of the MWIR and the LWIR bands using the self-assembled and SML QD groups, respectively. The growth strain-driven band structure engineering offers an alternative way to achieve dual-band photodetectors [19]. 

## 3. Material Growth and Device Fabrication Process

The multiple stacks of SML QD and self-assembled QD based dual band photodetector structure was grown on a semi-insulating GaAs (100) substrate using a V80H molecular beam epitaxy (MBE) system. A 3000 Å undoped GaAs buffer layer was first grown on the substrate, followed by the growth of a 1500 Å n+ doped GaAs bottom contact layer. Another 1000 Å undoped GaAs buffer layer was then grown on the bottom contact layer, followed by the growth of the 10 period SML QD layers. Each period of the SML QD layer consists of 10 repeats of the InAs 0.45 mL and 1 mL GaAs stacks. A 20 Å of Al_0.1_Ga_0.9_As and a 450 Å GaAs spacer layer were grown between the two adjacent SML QD layers as the separation layer. 

**Figure 2:**
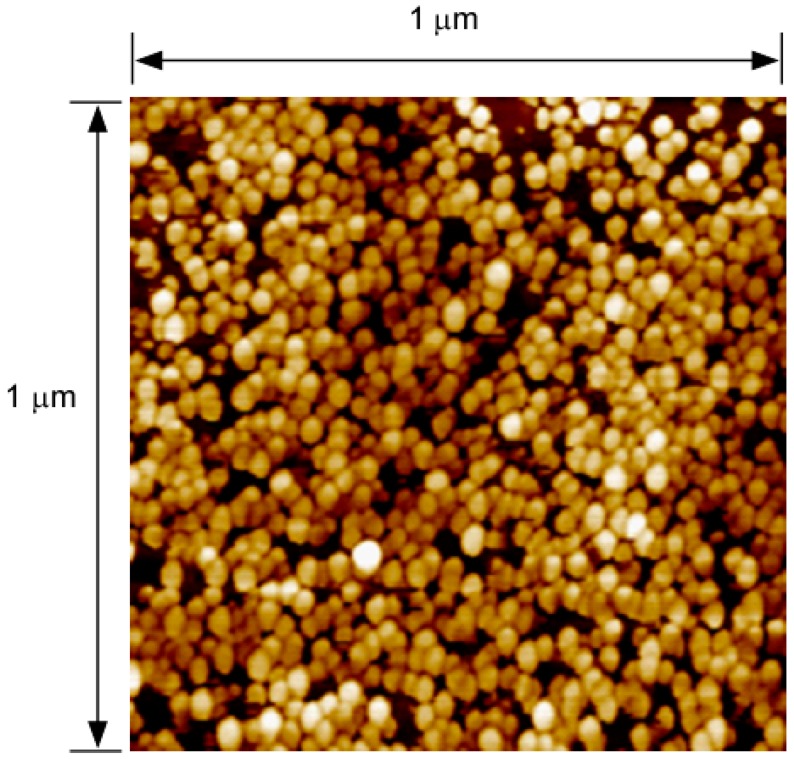
Atomic force microscopy (AFM) image of the QDs grown at the same growth conditions as the photodetector device. The SML QDs are uniform with a high density of ~ 4 × 10^10^/layer.

Figure 2 shows an atomic force microscopy (AFM) (Park Systems, Suwon, Korea) image of SML QDs grown under the same growth conditions as the photodetector device. The SML QDs are quite uniform with a high density of ~ 4 × 10^10^/layer.

After the SML QD growth, 10 periods of the self-assembled QD layers were grown on the SML QD layers. Each period of the self-assembled QD heterostructures consists of 1 nm In_0.15_Ga_0.85_As, 2 mL of InAs QDs, 6 nm In_0.15_Ga_0.85_As cap layer and 45 nm GaAs spacer layer. A 500 Å undoped GaAs buffer and a 0.1 µm n+ doped GaAs top contact layer were finally grown to finish the MBE growth. The doping level of the SML and the self-assembled QD regions were tuned to be approximately 2 electrons per dot. The SML and the self-assembled QD layers were grown at 485 °C. All the other buffer and contact layers were grown at 580 °C.

After the growth, the wafer is then processed into 250 µm-diameter circular mesas with top and bottom electrodes using standard photolithography, wet etching procedures, E-beam metal evaporation deposition, lift-off and thermal annealing processes [7]. The finished device was mounted on a copper plate and put into a liquid nitrogen (LN_2_) cooled dewar with a Zinc Selenide (ZnSe) IR window.

## 4. Device Measurement and Characterization

**Figure 3:**
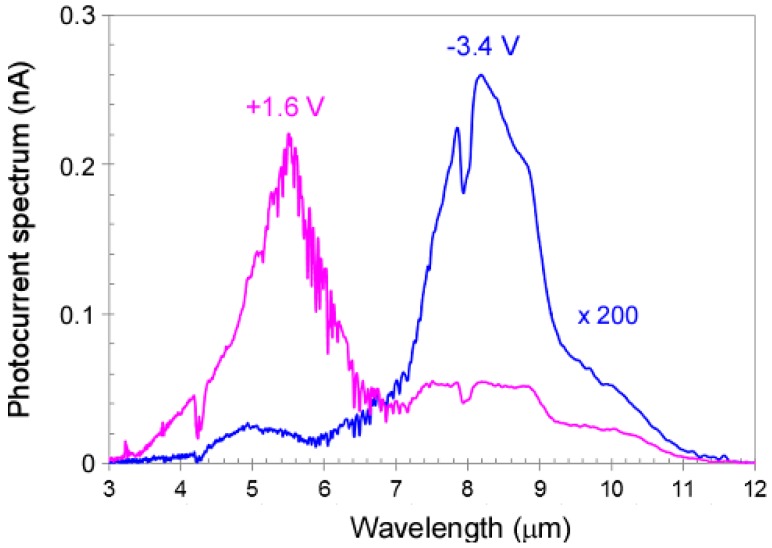
Photocurrent spectra of the dual-band QDIP under bias voltages of −3.4 V (blue trace) and +1.6 V (pink trace). The negative bias gives the LWIR band, whereas under the positive bias, the MWIR band dominates.

The spectral response of the dual-band QDIP was measured using a Fourier transform infrared spectrometer (FTIR) (Bruker, Leipzig, Germany) at 77 K. Detailed device characterization procedures have been reported [12]. Figure 3 shows the photocurrent spectra of the dual-band QDIP under bias voltages of −3.4 V (blue trace) and +1.6 V (pink trace). At the negative bias of −3.4 V, the QDIP only shows the IR photodetection from 6.8 µm to 10.5 µm with the peak wavelength of 8.2 µm. At +1.6 V, the photodetector covers the 4.0 µm to 6.8 µm wavelength regime with the peak wavelength of 5.5 µm. The photocurrent in the 6.8 µm to 10.5 µm spectral range is much larger than that in the 4.0 µm to 6.8 µm wavelength regime. 

**Figure 4:**
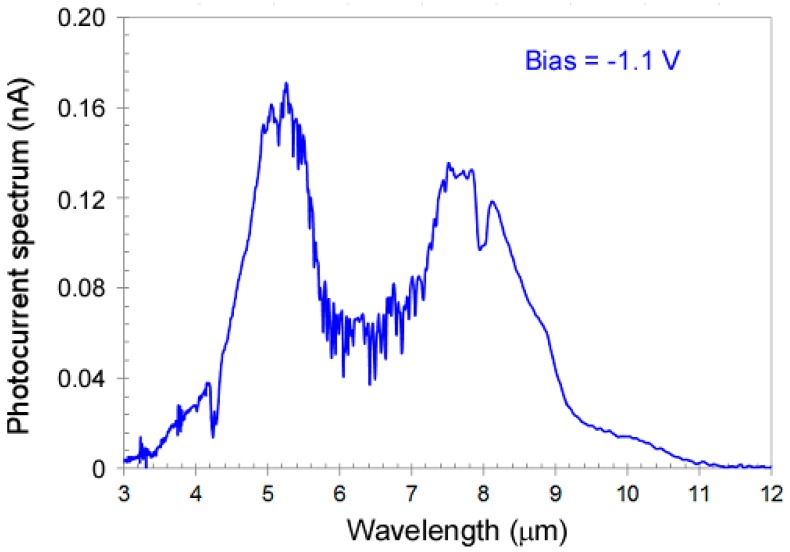
Photocurrent spectrum of the dual-band QDIP under bias voltages of −1.1 V. At this bias, the QDIP covers both the MWIR and the LWIR bands.

Figure 4 shows the photocurrent spectrum of the dual-band QDIP under the bias voltage of −1.1 V. Under this bias voltage, the QDIP covers both the MWIR and the LWIR band.

**Figure 5:**
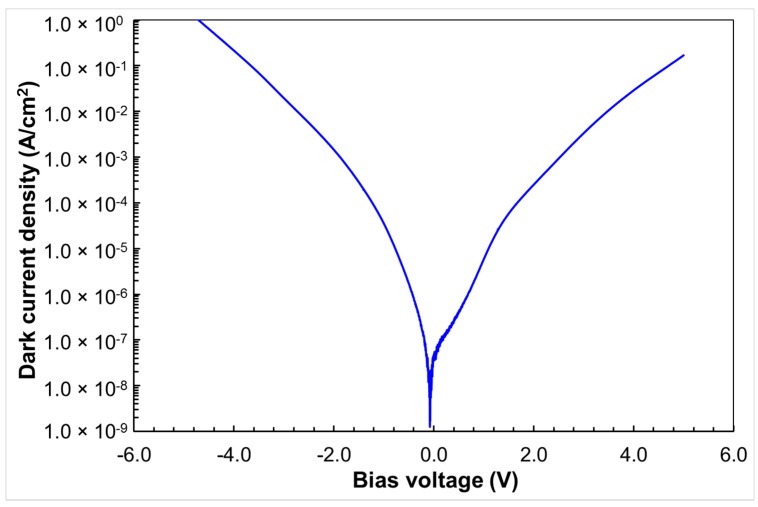
Dark current density (*J_d_*) of the dual-band QDIP under different bias voltages.

The dark current (*I_d_*) of the dual-band QDIP was measured using a Keithley source meter. Figure 5 shows the dark current density *J_d_* at different biases measured at the device temperature of 77 K. The noise current (*i_noise_*, in units of A/HzP^1/2^^P^) was characterized using a low-noise current preamplifier (Stanford Research Systems, Sunnyvale, CA, USA) and a fast Fourier transform (FFT) spectrum analyzer (Stanford Research Systems, Sunnyvale, CA, USA). To avoid 1/f noise contributions, the noise current (*i_noise_*) was determined at 597 Hz. 

The photoresponsivity ℜ for the MWIR and LWIR bands were measured using a (1000 K) calibrated blackbody source, with 3–5 µm and 8–12 µm band-pass filters. Since the detector bands are not exactly aligned with the band-pass filters, this would cut some IR signals and thus under-estimate the photoresponsivity of both bands. Nevertheless, we still observed the dual-band detection and voltage-controllable band tuning with good selectivity. The photocurrents were measured by modulating the filtered blackbody infrared source at a chopper frequency of 597 Hz, and the signal current was collected using a low-noise current preamplifier and an FFT spectrum analyzer. 

**Figure 6:**
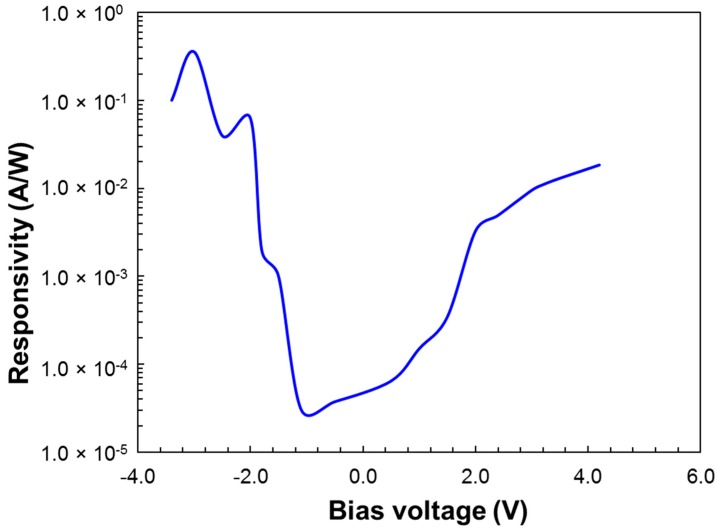
Photoresponsivity ℜ of the dual-band QDIP under different bias voltages.

Figure 6 shows the photoresponsivity ℜ of the dual-band QDIP under different bias voltages.

The photodetectivity D* can be calculated using:

(1) $$D^{*}=\frac{ℜ\sqrt{A}}{\sqrt{i_{noise}^{2}}}$$

where, *A* is the detector area, ℜ is the photoresponsivity, and *i_noise_* is the noise current.

**Figure 7:**
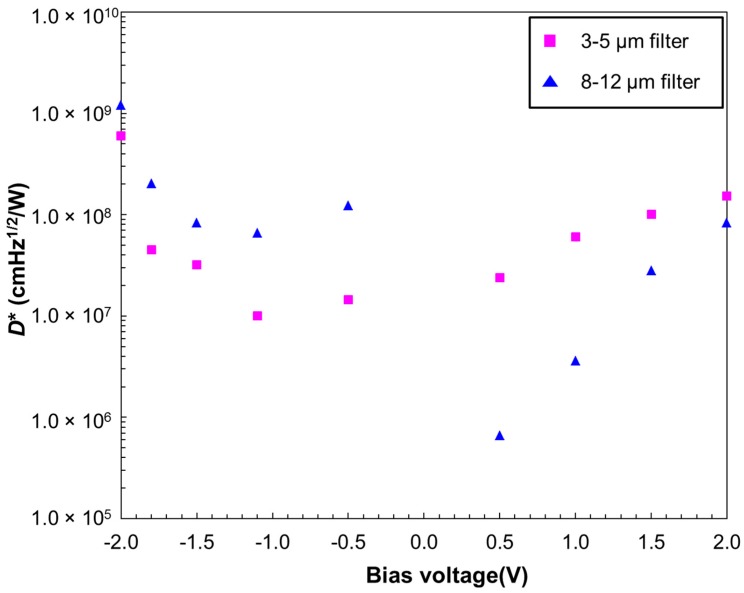
Calculated photodetectivity (D*) at different bias voltages with the 3–5 µm (square) and 8–12 µm (triangle) band pass filters. The 8–12 µm band has a higher D* at negative biases, whereas the photodetectivity D* of the 3–5 µm band is higher at positive biases.

Figure 7 shows the calculated photodetectivity (D*) at different bias voltages with the 3–5 µm (square) and 8–12 µm (triangle) band pass filters. The 8–12 µm band has a higher photodetectivity (D*) at negative biases, whereas the photodetectivity D* of the 3–5 µm band is higher at positive biases. The photodetectivity D* differences indicate good band selectivity by direct bias voltage control.

## 5. Conclusions

In conclusion, we report a dual-band QDIP based on vertically stacked SML and self-assembled QDs. The dual-band QDIP covers both the MWIR and LWIR spectral regimes. The dual-band QDIP also shows voltage-tunable detection band selection capability. By changing the photodetector bias voltages, one can set the detection band to be MWIR, or LWIR or both with high photodetectivity and low crosstalk between the bands.
